# Allicin Alleviates Inflammation of Trinitrobenzenesulfonic Acid-Induced Rats and Suppresses P38 and JNK Pathways in Caco-2 Cells

**DOI:** 10.1155/2015/434692

**Published:** 2015-02-02

**Authors:** Chen Li, Weijian Lun, Xinmei Zhao, Shan Lei, Yandong Guo, Jiayi Ma, Fachao Zhi

**Affiliations:** Guangdong Provincial Key Laboratory of Gastroenterology, Department of Gastroenterology, Nanfang Hospital, Southern Medical University, Guangzhou, Guangdong 510515, China

## Abstract

*Background*. Allicin has anti-inflammatory, antioxidative and proapoptotic properties. *Aims*. To evaluate the effects and investigate the mechanism of allicin on trinitrobenzenesulfonic acid-induced colitis, specifically with mesalazine or sulfasalazine. *Methods*. 80 rats were divided equally into 8 groups: control; trinitrobenzenesulfonic acid; allicin prevention; allicin; mesalazine; sulfasalazine; allicin + sulfasalazine, and mesalazine + allicin. Systemic and colonic inflammation parameters were analysed. In addition, protein and culture medium of Caco-2 cells treated with various concentrations of IL-1*β* or allicin were collected for investigation of IL-8, NF-*κ*B p65 P38, ERK, and JNK. One-way ANOVA and Kruskal-Wallis *H* test were used for parametric and nonparametric tests, respectively. *Results*. Allicin reduced the body weight loss of trinitrobenzenesulfonic acid-induced rats, histological score, serum TNF-*α* and IL-1*β* levels, and colon IL-1*β* mRNA level and induced serum IL-4 level, particularly in combination with mesalazine. In addition, 1 ng/mL IL-1*β* stimulated the P38, ERK, and JNK pathways, whereas pretreatment with allicin depressed this phenomenon, except for the ERK pathway. *Conclusions*. The inflammation induced by trinitrobenzenesulfonic acid is mitigated significantly by allicin treatment, particularly combined with mesalazine. Allicin inhibits the P38 and JNK pathways and the expression of NF-*κ*B which explained the potential anti-inflammatory mechanisms of allicin.

## 1. Introduction

Inflammatory bowel disease (IBD), comprising Crohn's disease (CD) and ulcerative disease (UC), is a nonspecific chronic disorder in the gut characterized by intestinal inflammation and mucosal damage. A systematic review of the incidence and prevalence of IBD indicated that the incidence of IBD in traditionally low prevalent regions, for instance, in Asia, has remarkably increased [1].

Current standard drug treatments for IBD include glucocorticosteroid, 5-aminosalicylates, sulfasalazine, immunosuppressor, infliximab, gene, and stem cell therapy. However, relapse and side effects, including allergy and granulocytopenia, remain during the whole treatment. Infliximab therapy appears to be safe and to have efficacious response and maintenance [2]; however, it is expensive and has some rare adverse effects, such as leukocytoclastic vasculitis and psoriasis [3, 4]. In addition, blocking one cytokine alone cannot inhibit or remove the coaction of multiple cytokines. Therefore, a much safer and more effective agent which is able to regulate upstream inflammatory pathways and subsequently suppress downstream cytokines is promising.

Garlic plays a crucial role in reducing infection, aging, and cardiovascular disease [5]. In addition, it has been reported that garlic may prevent colorectal tumor formation, reduce cholesterol and blood pressure, help to inhibit coagulation, and provide broad antioxidant activity to limit free radical damage [6]. An epidemiologic meta-analysis reported that a high intake of garlic may be associated with a protective effect against gastric cancer [7]. The intact garlic cloves contain alliinase and alliin in different compartments rather than allicin [8]. However, when the clove is cut or crushed, alliin and alliinase interact to form allicin which is responsible for the pungent odor of garlic and the antibiotic properties of garlic [6]. Allicin was first isolated and studied in the laboratory by Cavallito and Bailey in 1944 where it was found to have antibacterial properties [9]. It can induce apoptosis and the differential expression of proteins such as annexin I, galectin, and VDAC-1 in tumor cells [10]. It also modulates NF-*κ*B transcriptional and DNA binding activity, suppresses NF-*κ*B antiapoptotic target genes (Bcl-2, cIAP1/2) and inflammatory target genes (iNOS and COX-2), and consequently, as observed in human SW620 and HCT116 colon cancer cells, induces apoptosis [11]. As 1% of all cases of colorectal cancer occur in individuals with IBD [12, 13], it may be assumed that allicin may have a therapeutic effect on IBD.

CD is a T helper 1- (Th1-) mediated disease, with overproduction of IFN-*γ* and IL-12 [14, 15]. It is commonly thought that IL-12-mediated Th1 cell transmural colitis induced by trinitrobenzenesulfonic acid (TNBS) causes immunopathological human CD [16, 17]. To confirm the anti-inflammatory effects of allicin* in vivo*, the inflammatory index after administration of allicin and other proven anti-inflammatory drugs, namely, sulfasalazine and mesalazine, on TNBS-induced colitis in rats was investigated.

To study the mechanisms of the anti-inflammatory properties of allicin, the human colon adenocarcinoma cell line, Caco-2, was used. This is an ideal cell line to research the expression of proteins and functions of intestinal epithelial as this cell line imitates inflammatory status following stimulation with IL-1*β*. The phosphorylation of the P38, ERK, and JNK pathways was also accessed.

## 2. Materials and Methods

### 2.1. Induction of Colitis and Treatment of Rats

Eighty male Wistar rats (180–220 g, Experimental Animal Center of Southern Medical University, Guangzhou, China) were used in the study and acclimated under 12 h/12 h light-dark condition for 14 days. Animals were provided with* ad libitum* commercial rodent chow and had free access to drinking water. The study was approved by the Nanfang Hospital Animal Ethics Committee. On day 0, rats were anaesthetized with 2% pentobarbital sodium (Sigma-Aldrich, MO, USA), the catheter was carefully inserted into the colon (8 cm proximal to the anus), and TNBS mixture (diluted 1 : 1 in 100% ethanol) at 50 mg per kilogram body weight was slowly injected. For the control group (*n* = 10), the same volume of 50% ethanol was injected.

At 24 h after the enema, 60 rats were divided into 6 groups randomly (*n* = 10 each group) and were administered intragastrically with either saline; allicin (30 mg/kg, Cisen Pharmacy Company, Shandong, China); mesalazine (30 mg/kg, Ethypharm Industries, Saint-Cloud, France); sulfasalazine (100 mg/kg, Sine Pharmacy Company, Shanghai, China); allicin combined with mesalazine; or allicin combined with sulfasalazine at the same doses mentioned above. The final group (*n* = 10) had been treated with allicin (30 mg/kg) for 2 weeks prior to the enema and was then treated with saline (allicin prevention group). The control group (*n* = 10) treated with 50% ethanol alone was administered with saline. These procedures were repeated every 24 h.

All rats were weighed every other day and sacrificed on day 14 by anaesthetization and air-injection. The vitality and defecation of rats were observed. Blood and colonic tissue were processed as described below.

### 2.2. Macroscopic and Microscopic Assessment of Colonic Damage

The distal 8 cm of the rat colon and rectum was removed, opened longitudinally, washed, and then photographed. Following observation, full thickness specimens from inflamed tissue were fixed in 4% paraformaldehyde for 24 h, dehydrated, and embedded in paraffin. Tissue sections were cut at 4 *μ*m, stained with hematoxylin/eosin (H&E), and evaluated by 2 independent experienced pathologists in a blinded fashion, using a histological score ranging from 0 to 3. This index was based on the degree of epithelial damage and ulceration (0 = no; 1 = erosion; 2 = ulcer), ulcer and infiltration depth (0 = no; 1 = submucosa; 2 = muscularis; 3 = serosa), and edema and mononuclear cells infiltration (0 = no; 1 = mild; 2 = moderate; 3 = severe).

### 2.3. RT-PCR Analysis for IL-1*β* mRNA in Colon

Expression of colon mRNA coding for the cytokine IL-1*β* was quantified by RT-PCR as follows. The total RNA of the colon samples was isolated with Trizol (Takara Bio, Shiga, Japan) according to the manufacturer's protocol and used for the synthesis of first-strand cDNA using PrimeScript RT reagent Kit (Takara Bio, Shiga, Japan). The RT product was amplified by PCR with* Ex Taq* (Takara Bio, Shiga, Japan) and specific primers were as follows: IL-1*β* forward primer, 5′-TGA CCC ATG TGA GCT GAA AG-3′; reverse primer, 5′-GAA GAC AAA CCG CTT TTC CA-3′; *β*-actin forward primer, 5′-TGT CAC CAA CTG GGA CGA TA-3′; reverse primer, 5′-AAC ACA GCC TGG ATG GCT AC-3′. The lengths of the expected products were 241 bp for IL-1*β* and 195 bp for *β*-actin. The results were carried out 3 times and presented by intensity analysis and agarose gel electrophoresis.

### 2.4. Cell Cultures and Allicin Treatment

The Caco-2 cell line was obtained from the Cell Bank of the Chinese Academy of Sciences (Shanghai, China). Cells were grown at 37°C in 5% CO_2_ in high glucose DMEM (Gibco-BRC, NY, USA) supplemented with 100 *μ*g/mL streptomycin, 100 IU/mL penicillin (Genom, Zhejiang, China), and 10% fetal bovine serum (FBS) (Gibco-BRC, NY, USA). Cells were seeded at a density of 2 × 10^5^ cells/mL onto six-well tissue culture plates 48 h prior to assay.

After 24 h serum-free starvation, cells were treated with 0.1, 1, or 10 ng/mL of human recombinant IL-1*β* (Cell Signaling Technology, MA, USA) for 12 h, 24 h, or 48 h. Untreated Caco-2 cells served as control. 10 *μ*g/mL or 25 *μ*g/mL allicin was added with or without the IL-1*β* stimulation 2 h prior. The culture supernatant was collected and the cells were harvested for the following investigations.

### 2.5. MTT Assay

Approximately 2.5 × 10^3^ cells were seeded into 96-well culture plates and had been treated with or without IL-1*β* or allicin for 12, 24, or 48 h, respectively. After adding 3-(4,5-dimethylthiazol-2-yl)-2,5-diphenyltetrazolium bromide (MTT, Sigma-Aldrich, MO, USA) and the incubation, the absorbency at 490 nm was measured using microplate reader (Bio-Rad, CA, USA). The experiments were carried out for at least 3 times.

### 2.6. Western Blot Analysis

We separated nuclear and cytoplasmic protein of the harvested cells and then detected the levels of NF-*κ*B p65 (C22B4) subunit and histones 2A (D603A, 1 : 1000, Cell Signaling Technology, MA, USA) in the nucleus. The cells were also lysed in 200 *μ*L of lysis buffer (Beyotime, Jiangsu, China) with 1 mmol/L PMSF (Beyotime, Jiangsu, China) and phosphatase inhibitor cocktail (Cell Signaling Technology, MA, USA). Total protein was resolved on 12% SDS-PAGE gels and transferred to 0.45 *μ*m PVDF membranes (Millipore, Darmstadt, Germany). Western blot analysis was performed to determine cellular levels of p-P38 (D3F9), P38, p-ERK (D13.14.4E), ERK (137F5), p-JNK (81E11), JNK (56G8) (1 : 1000, Cell Signaling Technology, MA, USA), and *β*-actin (C4, 1 : 1000, ZSBIO, Beijing, China). Bands were visualized with secondary HRP-conjugated antibodies (1 : 3000, ZSBIO, Beijing, China) and the ECL system (Millipore, Darmstadt, Germany). Anti-H2A and *β*-actin were used to show equal sample loading. All the tests were carried out for at least 3 times.

### 2.7. ELISA Assay

Serum and cell culture medium were diluted with assay diluent. TNF-*α* (sensitivity: 5 pg/mL), IL-1*β* (sensitivity: 5 pg/mL), IL-4 (sensitivity: 0.2 pg/mL) and IL-10 (sensitivity: 1.5 pg/mL) levels in rats' serum and IL-8 (sensitivity: 7.5 pg/mL) level in cell culture medium were examined by commercial ELISA kits (eBioscience, CA, USA; R&D systems, MN, USA). Kits are specific for natural and recombinant rat TNF-*α*, IL-1*β*, IL-4, IL-10 and human IL-8, respectively.

### 2.8. Statistics

Experiments were performed at least three times. Data was processed and analyzed using the SPSS 13.0 software (Chicago, IL, USA). The results were expressed as mean ± standard deviation (SD). Parametric data were analyzed using one-way analysis of variance (ANOVA) followed by LSD-*t* or Dunnett T3 post hoc tests, as appropriate. The Kruskal-Wallis *H* test was performed to assess categorical data. The body weight was evaluated by covariance analysis. A *P* value of *P* < 0.05 was considered statistically significant.

## 3. Results

### 3.1. Allicin, Particularly in Combination with Mesalazine, Alleviates TNBS-Induced Colitis in Rats

**Figure 1:**
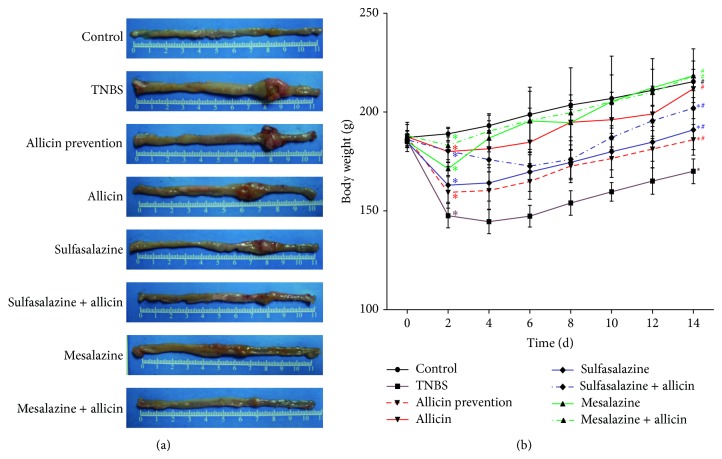
(a) Morphology of the colon in each group. TNBS induced a marked colon lesion, and, 14 days later, different treatments diminished the congestions and intumescences in varying degrees. Allicin combined with sulfasalazine or mesalazine reversed the damage of the colon. (b) The weight curve of rats. The day after the edema, rats lost weight significantly by TNBS inducing, even treated by drugs. At day 14, weights of control, allicin, mesalazine, and mesalazine + allicin groups had no significant difference. Each point represents mean ± SD of each group at certain days (*n* = 10 in control and mesalazine + allicin groups, *n* = 9 in sulfasalazine group, *n* = 8 in sulfasalazine + allicin group, *n* = 7 in allicin prevention and allicin groups, and *n* = 6 in TNBS and mesalazine groups). ^*^ *P* < 0.01 versus control group; ^#^ *P* < 0.01 versus TNBS group.

TNBS-induced colitis in rats displayed marked edema bleeding, intestinal mucosa thickening, and ulcers (Figure 1(a)). Intragastric gavage with allicin, sulfasalazine, or mesalazine for two weeks facilitated the healing of TNBS-induced colitis. Moreover, allicin combined with sulfasalazine or mesalazine reversed the damage of the colon. Rats in the allicin, mesalazine, and mesalazine + allicin groups showed less body weights loss compared with TNBS group at day 14 (Figure 1(b)). However, all the drugs did not alter the fatality rate of TNBS-induced rats significantly (*χ* ^2^ = 11.138, *P* = 0.133).

### 3.2. Allicin Histologically Improved the TNBS-Induced Colitis

**Figure 2:**
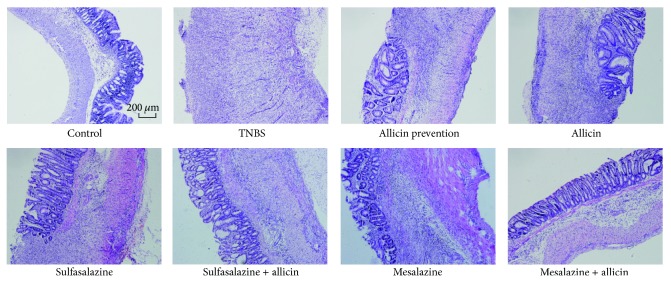
Histological photographs of H&E stained colon after 2-week treatments (100×). TNBS-induced rats had severe colitis with deep ulceration, transmural leukocyte infiltration, loss of goblet cells, and thickening of intestinal walls. Different treatments relieved colitis in various degrees. Sulfasalazine and mesalazine + allicin made the colons basically normal.

Control group colons showed minimal numbers of lymphocytes in the lamina propria and regularly arranged crypts. In addition, there was no inflammatory infiltration in the submucosa (Figure 2). The TNBS edema led to severe colitis with deep ulceration, transmural leukocyte infiltration, and loss of goblet cells. Two weeks after various treatments, considerable evidence of regeneration was seen. The Kruskal-Wallis *H* test was used to analyze the H&E staining score and the result was presented by mean rank (Table 1). The severer the inflammation was, the higher the rank was. There was a significant difference among the groups (*χ* ^2^ = 44.464, *P* = 0.000) with the mean rank of the control group being the lowest (mean rank = 6.06) whilst that of the TNBS with saline treatment group being the highest (mean rank = 57.75). Among the treatment groups, the rank of the mesalazine + allicin group was the lowest (mean rank = 22.30).

### 3.3. Allicin Combined with Mesalazine Downregulated Proinflammatory Cytokines and Upregulated Anti-Inflammatory Cytokines

**Figure 3:**
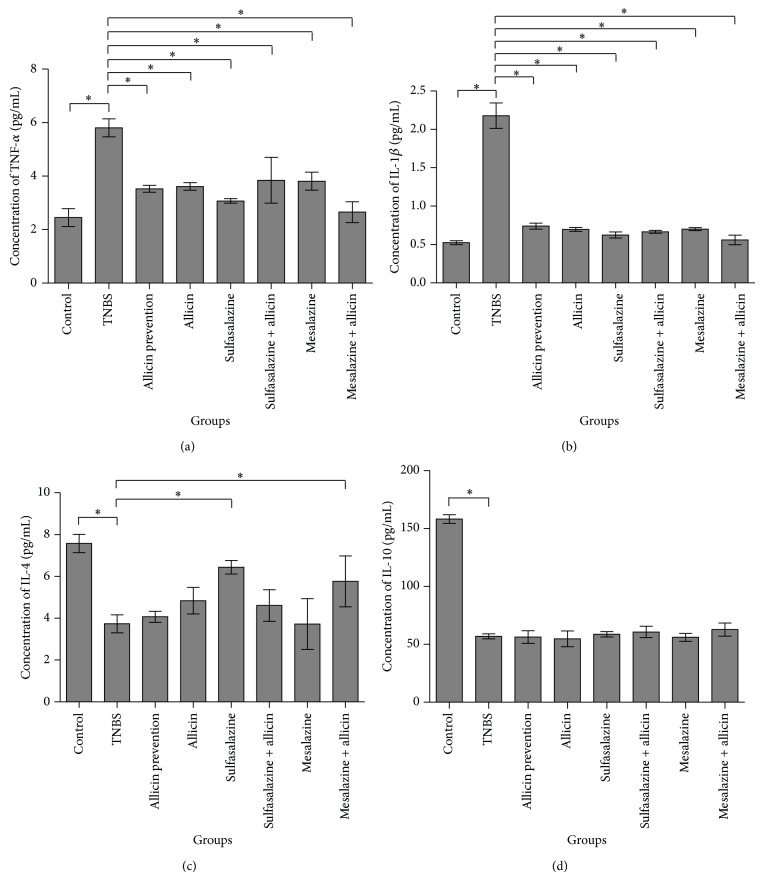
Levels of cytokines in the serum. Serum TNF-*α* and IL-1*β* levels of TNBS-induced rats were dramatically decreased by drug treatment, and mesalazine + allicin group was the lowest (a and b); sulfasalazine and mesalazine + allicin rose serum IL-4 level; however, these drugs did not alter serum IL-10 level (c and d). Data were presented as mean ± SD. ^*^ *P* < 0.05 versus TNBS group.

**Figure 4:**
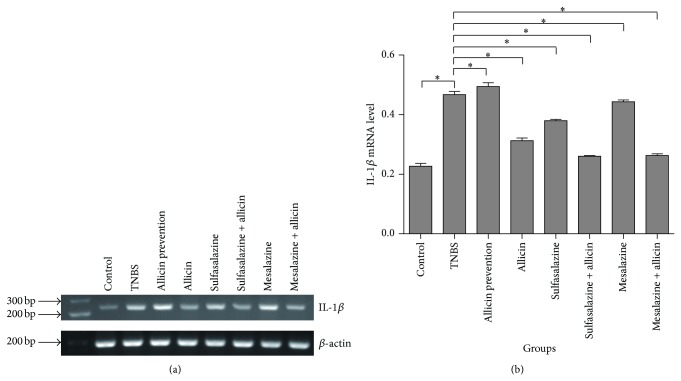
Expression of colon IL-1*β* mRNA level in rats. (a) Total RNA was isolated from colon tissue. Colon IL-1*β* mRNA levels were presented on an agarose gel. (b) The gel was carried out by densitometric and statistical analysis. Results were reported as means ± SD from 3 independent experiments. ^*^ *P* < 0.01 versus TNBS group.

Serum TNF-*α* and IL-1*β* levels were decreased in the treatment groups compared with that of the TNBS group (5.8050 ± 0.337, 2.1783 ± 0.166, resp.; *P* = 0.000) (Figures 3(a) and 3(b)). In particular, concentrations of them in the mesalazine + allicin groups were the lowest (2.6539 ± 0.389, 0.5605 ± 0.063, resp.), and there were no significant differences between the control group and the mesalazine + allicin group (*P* > 0.05). IL-*β* mRNA levels were also reduced significantly in the mesalazine + allicin group (*F* = 485.733, *P* = 0.000). Among the treatment groups, IL-*β* mRNA levels were notably low in sulfalazine + allicin and mesalazine + allicin, between which no significant difference was found (*P* = 0.71) (Figures 4(a) and 4(b)). Moreover, we also found that both the sulfasalazine and the mesalazine + allicin group significantly increased the IL-4 secretion after the edema compared to the TNBS group (6.4367 ± 0.328, 5.7637 ± 1.217, resp.; *P* = 0.000) but not that of IL-10 (Figures 3(c) and 3(d)).

### 3.4. Allicin Alleviates Nuclear NF-*κ*B p65 Expression and Inhibits the P38 and JNK Pathways in IL-1*β*-Treated Caco-2 Cells

**Figure 5:**
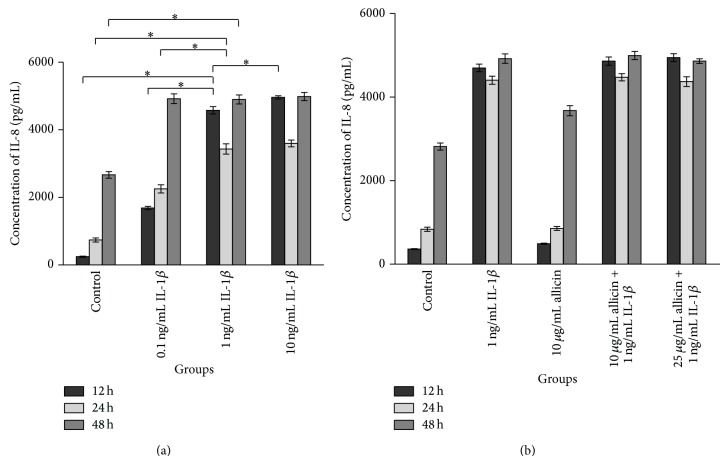
The influence of IL-1*β* and allicin on IL-8 in the supernatant of Caco-2 cells. (a) 1 ng/mL IL-1*β* significantly increased concentrations of IL-8 in the medium of Caco-2 cells at 12 and 24 h; (b) IL-8 level was not changed by allicin on IL-1*β*-induced Caco-2 cells.

**Figure 6:**
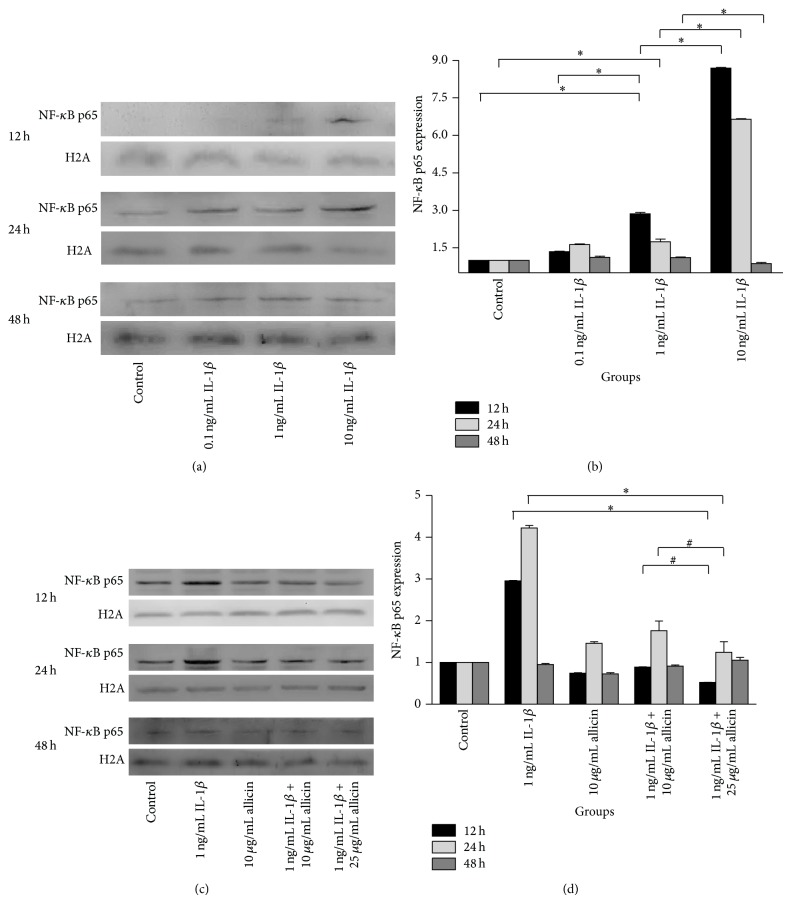
NF-*κ*B p65 was increased in IL-1*β*-induced Caco-2 cells (a and b): IL-1*β* induced the expression of NF-*κ*B p65 in nucleus of Caco-2 cells at 12 and 24 h. ^*^ *P* < 0.05 versus 1 ng/mL IL-1*β* group. Allicin influenced inflammation caused by IL-1*β* in Caco-2 cells (c and d): 25 ng/mL allicin decreased the expression of NF-*κ*B p65 in nucleus of IL-1*β*-induced Caco-2 cells at 12 and 24 h. ^*^ *P* < 0.05 1 ng/mL IL-1*β* group versus 1 ng/mL IL-1*β*  + 25 *μ*g/mL allicin group. ^#^ *P* < 0.05 1 ng/mL 1 ng/mL IL-1*β*  + 10 *μ*g/mL allicin group versus 1 ng/mL IL-1*β*  + 25 *μ*g/mL allicin group. Data were presented as mean ± SD and repeated 3 times.

In the MTT assay, treatment with 50 ng/mL allicin inhibited Caco-2 cell growth with 24 h incubation and this suppression of cell growth was enhanced with prolonged incubation and increased concentration of allicin (data not shown). Then we used ELISA to measure the levels of IL-8 and western blot to detect nuclear NF-*κ*B p65 in 0.1 ng/mL, 1 ng/mL, and 10 ng/mL IL-1*β*-treated Caco-2 cells at 12 h, 24 h, and 48 h. As shown in Figures 5(a), 6(a), and 6(b), IL-1*β* treatment promoted the secretion of IL-8 and NF-*κ*B p65 expression in the nucleus, which was enhanced by increased concentration of IL-1*β* at the 12 h and 24 h time points.

Allicin was given to investigate its anti-inflammation effect* in vitro*. It is shown that 10 and 25 *μ*g/mL allicin did not affect the level of IL-8 in the medium but downregulated the expression of nuclear NF-*κ*B p65 of IL-1*β*-treated cells at 12 h and 24 h time points (Figures 5(b), 6(c), and 6(d)).

**Figure 7:**
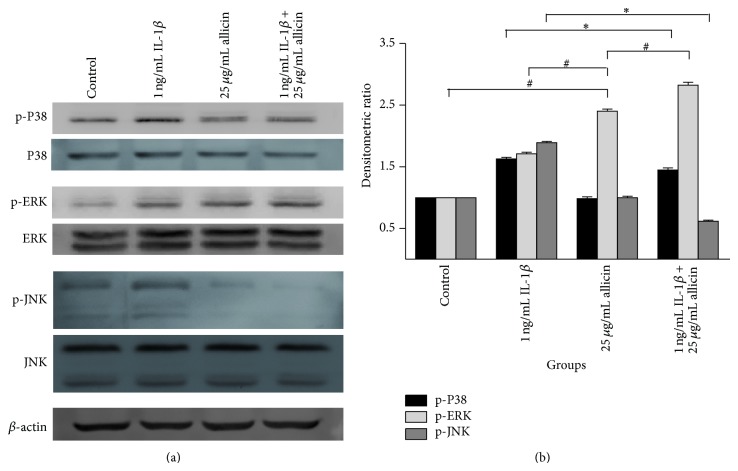
The change of P38, ERK, and JNK of allicin on IL-1*β*-induced Caco-2 cells. Results were repeated 3 times and presented by SDS-PAGE (a) and densitometric analysis (b). 25 *μ*g/mL allicin attenuated IL-1*β*-induced phosphorylations P38 and JNK pathways. Both 1 ng/mL IL-1*β* and 25 *μ*g/mL activated ERK pathway. ^*^ *P* < 0.05 1 ng/mL IL-1*β*  + 25 *μ*g/mL allicin versus 1 ng/mL (IL-1*β* p-P38 and p-JNK). ^#^ *P* < 0.05 versus 25 *μ*g/mL allicin (p-ERK).

Following the above experiments, 25 *μ*g/mL allicin was chosen as the appropriate concentration and 24 h as the appropriate treatment time point. The change of P38, ERK, and JNK in response to allicin treatment in IL-1*β*-induced Caco-2 cells was also investigated (Figure 7). In 1 ng/mL IL-1*β* stimulated Caco-2 cells, the P38, ERK, and JNK pathways were stimulated by phosphorylation. 25 *μ*g/mL allicin significantly inhibited the P38 and JNK pathways induced by IL-1*β*, whilst it did activate the ERK pathway with and without IL-1*β*.

### 3.5. The P38 and JNK Pathways Were Inhibited by Allicin

As observed in the above experiment, allicin could decrease the expression of nuclear NF-*κ*B p65, a downstream target of the mitogen-activated protein kinase (MAPK) pathways, in IL-1*β*-treated cells. To further study the mechanism of the anti-inflammatory effect of allicin, the change of P38, ERK, and JNK in response to allicin treatment in IL-1*β*-induced Caco-2 cells was investigated (Figure 6). In 1 ng/mL IL-1*β* stimulated Caco-2 cells, the P38, ERK, and JNK pathways were stimulated by phosphorylation. Whilst 25 *μ*g/mL allicin alone did not significantly inhibit the P38 and JNK pathways, it did activate the ERK pathway with and without IL-1*β*.

## 4. Discussion

TNBS-induced colitis is a widely used animal model for investigating the mechanism of acute or chronic colitis. It normally causes mucosal inflammation, intestinal ulcers, and histological damage and triggers colonic expression of the proinflammatory cytokines TNF-*α*, IL-1*β*, and IL-6 in 14 days after the TNBS administration [18]. In our study, we successfully established TNBS-induced colitis by enema and then treated rats with allicin, sulfasalazine, and mesalazine alone or in various combinations. Fatality rate, body weight, colon histological H&E staining scores, colon IL-1*β* mRNA levels, and serum cytokine levels were assessed in TNBS-treated rats. It is exciting to find that allicin alone or combined with mesalazine significantly improve the TNBS-induced colitis, which brings valuable clue for clinic treatment strategies of IBD.

Allicin, extracted from garlic, has anti-infection, antioxidative, antitumorigenesis, and proapoptotic properties [5, 6, 10, 11]. The focus of the current study was the anti-inflammatory effect of allicin, particularly when combined with sulfasalazine or mesalazine, and the possible mechanisms behind this process.

High levels of proinflammatory cytokines, such as TNF-*α*, IL-1, and IL-8 induce, amplify and prolong intestinal inflammation [19]. The potent cytokine IL-1 activates many immune and inflammatory cells. It is produced by various cell types including monocytes, neutrophils, and endothelial cells [20, 21]. In TNBS-treated rats, colonic mRNA levels of TNF-*α* and IL-1*β* are increased significantly [22]. Similarly, in the current study, TNBS-treated rats displayed the same tendency with an increase in serum TNF-*α* and IL-1*β* levels, as well as colon IL-1*β* mRNA levels, all of which were decreased following allicin treatment and in particular following treatment with allicin combined with mesalazine.

IL-4 and IL-10 have immunoregulatory and anti-inflammatory properties in gut immunology [23, 24]. In the current study, allicin did not alter serum IL-4 levels; however, sulfasalazine treatment and allicin combined with mesalazine significantly promoted them. But the serum levels of IL-10 were not altered by any of the treatment drugs, most likely because these drugs fell short of varying IL-10 levels. A research about the proinflammatory effect of allicin on mice with malaria infection, intraperitoneally injected with* P. yoelii* 17XL, also showed that it did not cause major changes of IL-10 levels of splenocytes supernatants.

Although the tendencies of different inflammatory indices were not accordant among the treatment groups in the current study, it could still be inferred that allicin relieves the inflammation of TNBS-induced colitis, particularly when combined with mesalazine. This might be because variant drugs regulate diverse inflammatory targets. The H&E score and serum TNF-*α* and IL-1*β* levels of sulfasalazine group were lower than sulfasalazine + allicin group, and serum IL-4 level of sulfasalazine group was higher than sulfasalazine + allicin group, possibly because the two compounds compete the inflammatory targets each other.

Caco-2 cell line was used in the* in vitro* experiments for it was more responsive to IL-1*β* compared to TNF-*α* or lipopolysaccharide (LPS) [25]. IL-8, the neutrophil chemoattractant and activator [23], is increased in diseased mucosa [26]. In addition, the level of IL-8 correlates with the macroscopic grade of local inflammation and neutrophil numbers in mucosal tissue, particularly in patients with UC [27]. Increased expression of NF-*κ*B is the key in the development of IBD. Stimulation of NF-*κ*B induces the phosphorylation, ubiquitination, and degradation of I*κ*B, which phosphorylates NF-*κ*B p65 subunit on Ser276, and then promotes nuclear translocation and subsequent DNA binding. Together, these processes promote downstream IL-1*β* and TNF-*α* expression which can then activate NF-*κ*B further, so that the inflammatory state develops gradually [28–30]. In the current study, IL-1*β* increased secretion of IL-8 and nuclear NF-*κ*B p65 expression with increased concentration and increased time. When the appropriate amount of allicin was added 2 h prior to IL-1*β* stimulation, IL-8 production was not significantly altered; however, nuclear NF-*κ*B p65 expression was suppressed by allicin, particularly at a high dose. As cytokines in the network interact with each other, IL-8 could be influenced by the change of IL-1*β* and TNF-*α* caused by the variable p65 submit translocation. These results further support an anti-inflammatory role for allicin, possibly via one of the MAPK pathways, as NF-*κ*B is the target of the MAPK pathways.

Three important MAPK pathways, P38, ERK, and JNK, were investigated in the current study. These pathways regulate diverse processes ranging from proliferation and differentiation to apoptosis [31]. Activation of P38 is characteristically and greatly increased in mucosal biopsies from patients with active IBD, followed by the activation of multiple proinflammatory cytokines [32]. Meanwhile, the JNK pathway plays crucial roles in regulating responses to various stresses and in the response of epithelial cells to stress conditions [33], neural development, inflammation, and apoptosis. IL-1 stimulation has been shown to strongly activate JNK [34] which in turn mediates the phosphorylation of c-Jun [33]. The present study showed that allicin downregulates the phosphorylation of P38 and JNK and the expression of NF-*κ*B p65 in Caco-2 cells, which were all elevated following IL-1*β* treatment (Figures 6(c), 6(d), and 7). It can therefore be concluded that allicin performs an anti-inflammatory role through reducing NF-*κ*B and suppressing the P38 and JNK pathways. The probable mechanism is allicin works on the kinase upstream, and then P38 and JNK regulate downstream target NF-*κ*B. A study focusing on diallyl disulfide (DADS) [35], a component of garlic, indicated that addition of 25, 50, 100, and 500 *μ*mol/L DADS to human colorectal cancer cells (HCT-15 cells) elevates ERK phosphorylation by 39, 52, 73, and 61%, respectively. Concurrently, there were no differences in ERK expression in cultures grown in the presence or absence of DADS. The addition of DADS also decreased the proportion of HCT-15 cells in the G1 phase and increased the proportion of cells in the G2M phase [36]. As allicin activated the ERK pathway in the current study, it is possible that the anti-inflammatory mechanism of allicin is via the mediation of apoptosis through activation of the ERK pathway.

## 5. Conclusions

In this study, we found that the TNBS-induced inflammation in rats is mitigated significantly by allicin treatment, particularly when combined with mesalazine. It is likely that allicin treatment, as observed by the decreased expression of NF-*κ*B, inhibits the P38 and JNK pathways whilst activating the ERK pathway and mediating apoptosis. However, the present research has limitation. Although allicin has therapeutic effect of experimental colitis, we do not know whether it is capable of curing patients with IBD since any animal model can rarely imitate the real situation of a patient. As current standard drug treatments for IBD often result in relapse and adverse side effects or are extremely expensive, allicin, as a regulator of upstream inflammatory pathways and the subsequential suppression of downstream cytokines, may be a much safer and effective agent in the treatment of IBD after a rigorous clinical trial.

## Figures and Tables

**Table 1:** Histological scores of colon tissues ($\overset{-}{x}\pm S$).

Groups	Score	Mean rank order
Control (*n* = 10)	0.22 ± 0.441	6.06
TNBS (*n* = 6)	5.83 ± 0.753	57.75
Allicin prevention (*n* = 7)	4.29 ± 0.756	45.14
Allicin (*n* = 7)	4.29 ± 1.113	44.50
Sulfasalazine (*n* = 9)	2.22 ± 0.972	23.33
Sulfasalazine + allicin (*n* = 8)	3.38 ± 1.408	35.50
Mesalazine (*n* = 6)	3.33 ± 0.816	34.75
Mesalazine + allicin (*n* = 10)	2.10 ± 1.197	22.30

χ^2^		44.464
*P*		0.000

The Kruskal-Wallis *H* test was performed to assess the histological scores.
